# Hospital Meetings, &c.

**Published:** 1901-06-22

**Authors:** 


### HOSPITAL MEETINGS, &c.

### KING'S COLLEGE HOSPITAL.

#### Festival Dinner.

The Archbishop of Canterbury presided at the annual dinner of King's College Hospital, held at the Clothworkers' Hall, on the 12th instant. Proposing the toast of "Success to the Hospital," His Grace said he knew of no institutions which more deserved that they should wish them prosperity than those great hospitals where the comfort and welfare of the sick was always being studied and provided for. Next to the preaching of the Gospel was, he thought, the service of those who devoted their lives to the alleviation of suffering and the healing of disease. Medical science had been making such progress during the past few years as never before in the history of the world. Men would look back with wonder, in years to come, on the advance made in the last half-century: to the discoveries of Lord Lister; to the use of antiseptics; and they owed much to those who were still engaged in discovering how diseases were spread. There were possibilities of doing now what was never so much as thought of 100 years ago, and King's College Hospital had its share in the claim to recognition for these great benefits to mankind about which it was difficult to moderate our language. The medical profession was, in its very essence, one of the very noblest professions, enlisting at once the greatest powers of the intellect and of the heart.

Lord Dillon replied to the toast, and expressed the conviction that the hospital had in no way fallen out of the very front rank of such institutions.

The Hon. W. F. D. Smith, M.P., proposed "The National Services," the Bishop of Bristol replying for "The Church," Mr. Aldred Rowden, K.C., for "The Law," and Lieut.-Colonel Fellows for "The Imperial Forces."

The Rev. Canon Ainger, Master of the Temple, proposed "The Medical Staff of the Hospital," observing that they had on the staff now, as from the beginning, men of European reputations. He was glad to know that, unlike another institution with which they were all acquainted, they found the greatest benefit from having members of the staff on the governing body, and that the most complete harmony prevailed. In the co-operation of the staff they found assistance in the management and conduct of every detail.

Dr. Ferrier replied, and emphasised what had been said regarding the perfect harmony subsisting between the medical staff and the Board.

Mr. Chas. Awdry, the Treasurer, in proposing the health of "The Chairman," said their debt at the end of the year was £2,019, and he was happy to be able to announce that the subscriptions and donations resulting from that gathering amounted to £2,109, this being within £600 of the exceptionally good figure reached last year. He referred with regret to the absence, through illness, of their warden, the Rev. N. Bromley, and hoped the news they would be able to send him of the result achieved that evening would do him real good. Their annual expenditure was close on £20,000, and towards that their regular income did not go nearly far enough. In addition they had, at the suggestion of the Prince of Wales's Fund, adopted a reform which would cost them some £400 a year—the supply of tea, sugar, and butter to their patients. It was a move in the right direction, and he was sure their friends would see they did not suffer for it He also reminded all friends that the Convalescent Home at Hemel Hempstead was £1,600 in debt, and that they were incurring a further expenditure there of £2,000, and concluded with an earnest appeal for continued help, coupled with thanks to the Chairman for his very valuable assistance.

### NORTH LONDON CONSUMPTION HOSPITAL.

The Right Hon. H. H. Asquith, K.C., M.P., presided at the Festival Dinner of the North London Hospital for Consumption and Diseases of the Chest at the Hôtel Cecil on the 14th instant.

After the customary loyal toasts had been duly honoured, Dr. Blake Odgers, K.C., proposed "The Houses of Parliament," this being responded to by Mr. E. Brodie Hoare, M.P. The Chairman then gave the toast of the evening, "Prosperity to the North London Hospital for Consumption." He said: I suppose it would be difficult for the most ingenious orator to discover any new argument wherewith to elicit sympathy and support for our great Metropolitan hospitals. I, at any rate, shall abstain from entering on those general considerations; not only because they are of course familiar to all assembled here, but for the better reason that the particular charity in whose interests we are gathered is one which is battling against a particular form of disease which has been in times past a very scourge, and has visited with particular virulence the people of this country. And it is battling against this form of disease by means from which, although our experience is as yet somewhat short, we believe we may confidently anticipate the most useful and the most beneficent results. Knowledge in surgery has advanced by leaps and bounds in recent times, yet the sister art of medicine in its treatment of disease has not made the same progress, although diagnosis is far more accurate than was formerly the case, and although fields which used to be obscure and untravelled are being explored and made known. As to the reason of this I am not competent to express an opinion. It may be that as in other matters even at the moment when we think we have reached the stationary stage, we are perhaps on the eve or even on the morrow of discoveries which will result in revolutionary changes. During the last twelve months, for instance, we have had disclosed to us an extraordinary development with relation to a particular form of malady, malaria, against which for ages medical science has fought in vain, and under which very large tracts of the world have suffered. Now, largely owing to the zeal and courage of English doctors, we know that this disease is dependent not so much upon climate or soil, but upon the presence of a particular form of fly or mosquito. It seems as though if we can banish that agent for the transmission of the poison of malaria we shall banish one of the most intractable and widespread enemies of the human race. But if that is the case with malaria, may not some similar development of knowledge be possible in connection with consumption. In 1834, when Sir Robert Peel was summoned by William IV., he travelled post haste from Rome to London, and it has been calculated that he accomplished the journey in the same time that it had taken the Emperor Adrian 1,700 years before. But at that very time the Manchester railway was already in operation; the new era which has almost annihilated distance had already dawned! May it not be the same with consumption? We know that in days gone by this disease was regarded as the most widespread of disorders, and the persons suffering from it were looked upon as doomed, and were carefully immured, closely shut up away from contact with the fresh air. But the change of system now must be seen to be realised. Anyone now going to the North London Hospital will see the victims of what used to be considered an irremediable disaster, lying or sitting in open-air wards and balconies, with the air of heaven playing all round them, spending their days and nights almost under the canopy of heaven, and presenting as great a contrast to the doomed victims of past days as it is possible to conceive of. There are, I believe, 100 patients at any given time subject to this treatment. For some few years past they have been treated in the open-air wards with the windows open; and since June last there have been balconies outside the building which, with the exception of a roof to keep off the wet, are absolutely open. For six months there have been there 24 patients of both sexes, day and night, not only without any aggravation of their malady, but deriving healthy stimulus and curative influences from their surroundings. I suppose our experience has not been long enough or wide enough to arrive at dogmatic conclusions, but some of the figures obtained are remarkable. Of the patients so treated during the last twelve months, and suffering from actual phthisis, no less than 70 per cent. have been discharged, either much improved—that is, practically free of the disease or so distinctly improved so that they can return to their normal avocations. But these figures have to be discounted in a way to emphasise their significance, by the fact that many of the patients had been brought to the hospital when the disease had already reached an advanced stage. If they had come during its primary stages when the disease was confined to the apex of one lung only, they would have had a far better chance. Our figures show that of this class 94 per cent. were discharged either much or distinctly improved. But even that does not represent the full measure of value of this mode of treatment. However excellent our appliances, however great the skill of the medical staff, and the devotion and zeal of the nurses, we are dealing with patients who belong to the poorer classes, are ill-nourished, and come from bad and insanitary surroundings. They are obliged to be under the treatment for a limited space of time only. Six weeks is the term, or under exceptional circumstances this might be extended to nine or at most to twelve weeks. This, however, is not enough. We cannot really hope to attain the best results unless those weeks are extended to months, or at any rate largely increased. There is another thing to be borne in mind. We have to deal with the patients on what may be called an economic system. They cannot be fed as you would feed members of the wealthy classes, because they have to return to their far more meagre and harder fare. So while feeding them well this has to be borne in mind. There cannot be a doubt that if the best results are to be achieved by the new system the patients must receive its full benefits for a longer time and under ampler dietetic conditions. However, I have said enough to show that the new departure is full of promise and full of hope. Cannot the provision be made more adequate? The institution has made steady progress. It was opened 40 years age with 40 beds. These were increased before 1899 to 80; and since 1899, when the new balconies were opened, the 80 has developed to 100, at an additional cost of £1,500. Last year there were nearly 500 patients passing through the wards, while very nearly 4,000 derived benefits as out-patients. But of the applications for admission, something like 60 per cent. have to be refused for want of room. There are 150 at the present time waiting for admission, so that the problem the committee have to meet is twofold, and they appeal to all who can help to provide for the additional expenditure caused by the opening of the 20 new beds in the balconies in 1899, and to make good the gap between the £6,700 of expenditure and the £6,200 which is all they can count on as regular income. And, in addition, the Governors have resolved to increase the present maximum of 100 beds to 130 by extensions costing some £12,000. Is it not possible to accomplish this? Even now when so much has been done to diminish the scourge of consumption, we are told that no less than one in seven of the population of this country is subject to it. This hospital offers relief to the poor who cannot afford to go to the high air of Switzerland or the noted health resorts of the Continent. I appeal to you to provide the committee with complete relief from anxiety with regard to the cost of the extension already made, with funds for the extension contemplated, and with an income that will admit of a longer stay in the institution, and so help to arrest the spread of one of the most terrible scourges of the country.

Mr. ALFRED HOARE, the Treasurer, and Mr. B. A. LYON, chairman of the committee, responded, and it was announced that promises had been received of £6,000 towards the £12,000 required for the extension, and donations and subscriptions of £2,486 towards the general funds.

